# A Novel Synthesis of Quasi-Chebyshev Ultra-Wideband Bandpass Filter Using *N*th Order Stub Loaded Coupled-Line Resonator

**DOI:** 10.3390/mi14101874

**Published:** 2023-09-29

**Authors:** Muhammad Abdul Rehman, Sohail Khalid, Bilal Mushtaq, Mueen Uddin, Jawaid Iqbal, Maha Abdelhaq, Raed Alsaqour

**Affiliations:** 1 Department of Electrical Engineering, Riphah International University, Islamabad 45210, Pakistan; s.khalid@riphah.edu.pk (S.K.); bilalmushtaq88@outlook.com (B.M.); 2 College of Computing and Information Technology, University of Doha for Science and Technology, Doha 24449, Qatar; 3 Faculity of Computing, Riphah International University, Islamabad 45210, Pakistan; jawaid.iqbal@riphah.edu.pk; 4 Department of Information Technology, College of Computer and Information Sciences, Princess Nourah Bint Abdulrahman University, P.O. Box 84428, Riyadh 11671, Saudi Arabia; msabdelhaq@pnu.edu.sa; 5 Department of Information Technology, College of Computation and Informatics, Saudi Electronic University, Riyadh 93499, Saudi Arabia; r.alsaqor@seu.edu.sa

**Keywords:** bandpass filter (BPF), short-circuit stub, coupled lines

## Abstract

This paper presents a novel synthesis of a quasi-Chebyshev *N*th order stub-loaded coupled-line ultra-wideband bandpass filter. A unit element of a proposed filter topology consists of two short-circuited stubs loaded at the edges of coupled lines. A distributed equivalent circuit model of a proposed topology is extracted and used to acquire a generalized filtering function. The extracted filtering function is of rational form. The denominator of the filtering function causes a mismatch with Chebyshev type-I polynomials. For conventional narrowband filters, the denominator term can be neglected because of the close vicinity of band-edge frequencies; however, for the ultra-wideband filter response, the factor in the denominator cannot be neglected and hence requires a new mathematical procedure to compensate for the effect of the frequency-dependent term in the denominator. The electrical parameters are calculated using the proposed synthesis and used to design an ideal filter topology on ADS. To validate the proposed design procedure, fabrication is performed on a high-frequency substrate. The proposed filter is miniaturized in size and has good out-of-band performance. The simulated and measured results provide good agreement.

## 1. Introduction

The increasing demand for miniaturized, cost-effective, and sophisticated wireless systems has driven the development of highly integrated multi-functional microwave circuits, particularly emphasizing passive microwave components. In response to this evolving landscape, there is a compelling need for the creation of a filter characterized by two key attributes: high selectivity and an expansive stopband. Such a filter not only ensures the reliable transmission of signals but also effectively mitigates the distortions induced by electromagnetic interference. Recent advancements have introduced several distinct methodologies for designing wideband bandpass filters. In the work delineated in [1], a wideband bandpass filter (WB-BPF) with exceptional selectivity is presented, employing the dual-path signal interference technique. It is noteworthy, however, that this wideband bandpass filter, while achieving commendable electrical performance, exhibits relatively substantial circuit dimensions. Furthermore, an alternative design for a wideband bandpass filter was proposed in [2], involving the utilization of a ring resonator and an open stub-loaded step impedance resonator. Additionally, ref. [3] introduces an innovative multimode resonator (MMR) that comprises three pairs of coupled lines loaded with stubs, aiming to provide multiple resonating modes for an ultra-wideband bandpass filter. Nevertheless, it should be acknowledged that this introduced filter is constrained by a sizable circuit footprint. An alternate approach to designing an ultra-wideband bandpass filter, as delineated in [4], involves the application of an interdigital coupled line combined with stub-loaded transmission lines. It is essential to note that filters proposed in this context possess a compact circuit size, albeit with certain compromises in terms of their electrical performance. In [5], a wideband bandpass filter is introduced utilizing a multimode split ring resonator within a waveguide cavity. Nevertheless, it remains a challenge to design a WB-BPF without resorting to complex design procedures while simultaneously achieving excellent electrical performance. A more straightforward design of an ultra-wideband bandpass filter with commendable electrical performance was proposed in [6]. This design entails the utilization of short-circuited stubs connected with a uniform impedance resonator. The favorable electrical performance is attributed to its multimode response; however, it results in a significantly large circuit size. In [7], a multilayer structure is employed to design WB-BPF, but the design procedure is intricate. Another approach for WB-BPF design, as detailed in [8], involves the use of a split ring resonator with rectangular stubs. However, this design compromises bandwidth and electrical performance. The development of an ultra-wideband bandpass filter (UWB-BPF) using the conventional method is presented in [9], where inductively compensated parallel coupled lines (ICPCL) are employed. The resulting filter is compact in size but experiences a compromise in electrical performance. Ref. [10] combines interdigital input/output coupled lines with multiple-mode resonators (MMR) to obtain UWB filters. Furthermore, for strong coupling under the input/output ports, a defective ground structure is etched as described in [11,12]. However, filters based on MMR exhibit two glaring flaws: poor passband return loss and low-frequency selectivity. Additionally, refs. [13,14] focused on the use of multilayer, microstrip to conductor-backed coplanar waveguide (CB-CPW) transitions for designing UWB BPFs, although the resulting filters are approximately one wavelength in size. On the other hand, the slot line design excels in wideband common-mode suppression, as mentioned in [15]. While achieving an ultrawideband (UWB) balanced filtering response due to high coupling between the feed line and the multiple modes of the slotline, regulating coupling coefficients proves to be a challenge. Ref. [16] presents a high-order quasi-absorptive bandpass filter achieved through the use of quarter-wavelength short circuit stubs shunt connected with parallel coupled transmission lines. A direct synthesis approach for a composite stub-loaded UWB filter is proposed in [17]. In [18], an ultra-wideband bandpass filter (UWB-BPF) designed using a coplanar waveguide (CPW) demonstrates excellent electrical performance. However, it is important to note that the filter’s size is relatively large. Another approach to designing a wideband bandpass filter with a compact size and excellent electrical performance is detailed in [19]. This method involves the use of substrate-integrated waveguide (SIW), although the design and fabrication process can be intricate. In [20], an innovative bandpass filter (BPF) that employs spoof surface plasmon polaritons (SSPPs) within a compact folded slotline configuration is introduced. This proposed filter excels in electrical performance and power handling, although concerns regarding circuit size persist. Numerous design techniques have been documented in the literature for the development of Wideband Bandpass Filters (UWB-BPF) [21,22,23,24,25]. Nevertheless, these approaches are often plagued by issues such as substantial circuit size, intricate fabrication processes, and elevated costs. In response to these challenges, various synthesis methods have been proposed as potential solutions for UWB-BPF design [17,26,27,28,29,30]. These synthesis techniques typically involve the derivation of generalized parameter values employing a Chebyshev-type filtering mechanism. It is worth noting, however, that the utilization of a Chebyshev-type filtering mechanism may not be universally applicable, as it is not valid for all arbitrarily powered rational filtering functions. Furthermore, the filtering function generated by this approach adheres to a specific periodic pattern, limiting its adaptability to other filtering function types. Therefore, a synthesis method that overcomes these limitations is imperative. Concurrently, previous research endeavors in the realm of UWB BPF development have primarily centered around the utilization of full-wave electromagnetic (EM) simulators and optimization tools. Traditional filter theory, rooted in the concept of narrowband filters, is inherently unsuitable for the development of UWB filters [31,32,33]. Given the inherent constraints associated with these methods and the unique characteristics of UWB filters, the development of an accurate synthesis theory for UWB bandpass filters has become more pivotal than ever before.

The synthesis method in this paper involves the development of a novel ultra-wideband (UWB) bandpass filter. This synthesis offers numerical accuracy, an optimal solution, and a comprehensive understanding of the filter. By obtaining the generalized filtering function through the equivalent circuit of a filter, this synthesis approximates the filtering function as a quasi-Chebyshev function. This approach enables the determination of parameters to achieve increased selectivity and equal ripple response, addressing the limitations associated with previous design methods. In light of the constraints and challenges posed by existing methodologies, the pursuit of accurate synthesis theory for UWB bandpass filters is now more critical than ever before in the context of the evolving wireless communication landscape.

## 2. Proposed Synthesis for UWB-BPF

**Figure 1:**
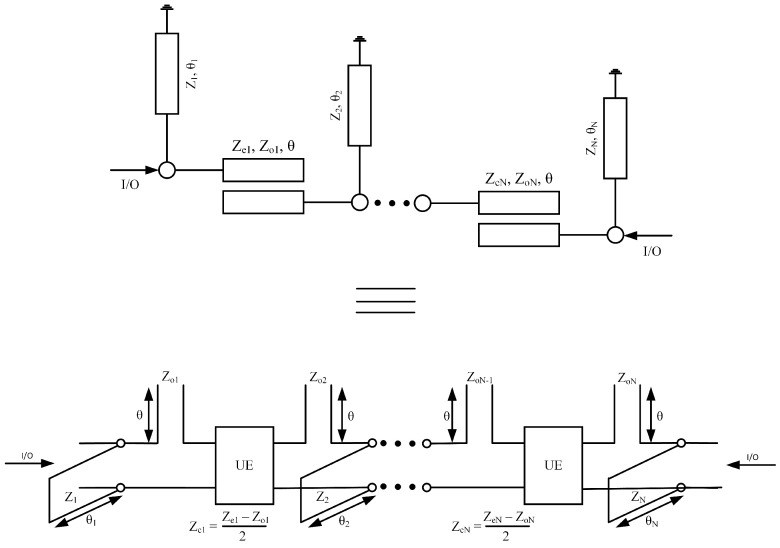
Schematic of *N*th order UWB-BPF with its equivalent circuit model.

Figure 1 shows proper circuit topology consisting of a *N*th order ultra-wideband bandpass filter with its distributive equivalent circuit model. Quarter wavelength coupled lines with loaded short-circuit stubs are used to design the proposed filter. The even and odd-mode characteristics impedance of coupled lines is $z_{e}$ ohm, $z_{o}$ ohm, whereas the characteristics impedance of open-circuit stubs is z. The distributed equivalent circuit model is used to extract the filtering function. Equation (1) shows the transfer matrix of the short-circuited stub’s shunt connected to the coupled lines.

(1) $$\begin{array}{c} \left[T\right]=\prod_{q=1}^{N}\left(\begin{array}{cc} 1 & 0\\ \frac{1}{jz\tan(\theta )} & 1 \end{array}\right)\times \prod_{q=1}^{N-1}\left(\begin{array}{cc} \cos(\theta ) & jz_{c}\sin\left(\theta \right)\\ \frac{j\sin(\theta )}{z_{c}} & \cos(\theta ) \end{array}\right)\times \prod_{q=1}^{N+1}\left(\begin{array}{cc} 1 & \frac{-jz_{o}}{\tan(\theta )}\\ 0 & 1 \end{array}\right) \end{array}$$

After determining the transfer function using Equation (1), the transmission coefficient $S_{21}$ is calculated using the known relationship illustrated in Equation (2). From the transmission coefficient $S_{21}$ [23], the filtering function can be extracted using Equation (3).

(2) $$\begin{array}{c} S_{21}=\frac{2}{A+B+C+D} \end{array}$$

(3) $$\begin{array}{c} {\left|S_{21}\left(j\omega \right)\right|}^{2}=\frac{1}{1+\varepsilon^{2}G_{N}^{2}\left(\theta \right)} \end{array}$$

Here, $G_{N}\left(\omega \right)$ indicates the filtering function of the *N*th order, and ε is the ripple level. The derived filtering function of the proposed filter is of the order (N=4).

(4) $$G_{N}\left(\theta \right)=\left\{\begin{array}{c} A_{1}\cos^{N}\left(\theta \right)-A_{2}\cos^{\left(N-2\right)}\left(\theta \right)+A_{3}\cos^{\left(N-4\right)}\left(\theta \right)......A_{N}\cos^{\left(0\right)}\left(\theta \right)\;for\;N=Even\\ B_{1}\cos^{N}\left(\theta \right)-B_{2}\cos^{\left(N-2\right)}\left(\theta \right)+B_{3}\cos^{\left(N-4\right)}\left(\theta \right)......B_{N}\cos^{\left(1\right)}\left(\theta \right)\;for\;N=Odd \end{array}\right.$$

.

### 2.1. Synthesis of 4^th^ Order UWB-BPF

**Figure 2:**
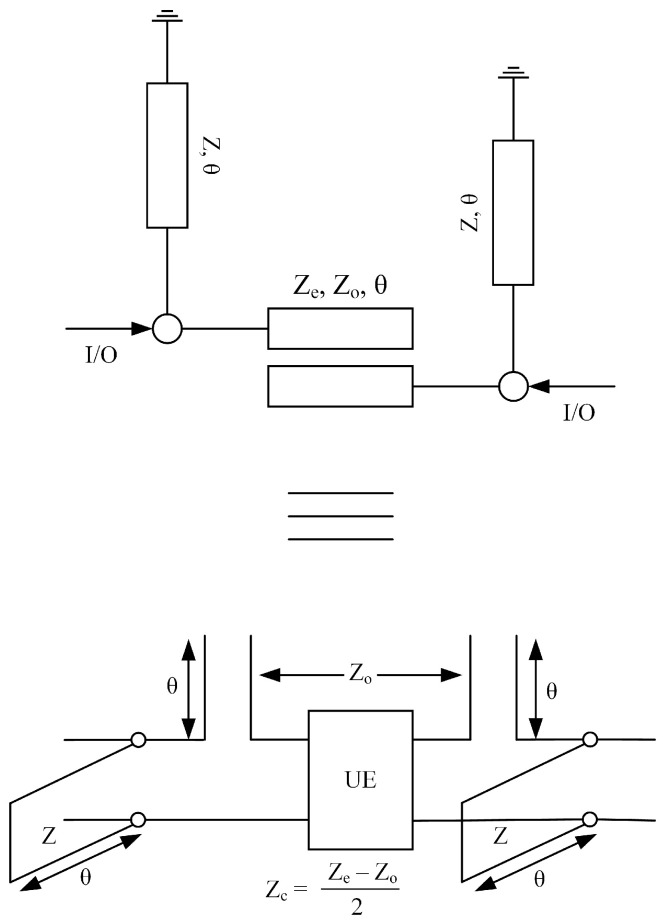
Schematic of 4th-order UWB-BPF with its equivalent circuit model.

In this section, a synthesis of fourth-order UWB-BPF is presented. Figure 2 shows the schematic model of the proposed fourth-order UWB bandpass filter with its equivalent circuit model. A single-stage quarter-wavelength coupled line with two loaded short-circuit stubs is used to design the proposed filter. The even- and odd-mode characteristics impedance of the coupled lines is $z_{e}$ ohm, $z_{o}$ ohm, whereas the characteristic impedance of the short-circuited stub is z. The filter’s overall transfer matrices are found by simply multiplying all the cascaded sections.

(5) $$\begin{array}{c} \left[T\right]=\prod_{q=1}^{2}\left(\begin{array}{cc} 1 & 0\\ \frac{1}{jz\tan(\theta )} & 1 \end{array}\right)\times \left(\begin{array}{cc} \cos(\theta ) & jz_{c}\sin\left(\theta \right)\\ \frac{j\sin(\theta )}{z_{c}} & \cos(\theta ) \end{array}\right)\times \prod_{q=1}^{2}\left(\begin{array}{cc} 1 & \frac{-jz_{o}}{\tan(\theta )}\\ 0 & 1 \end{array}\right) \end{array}$$

The filtering function $G_{4}\left(\theta \right)$ of the proposed single-stage stub loaded coupled line structure is found using Equation (3).

(6) $$G_{4}\left(\theta \right)=\frac{\alpha \cos^{4}\left(\theta \right)+\beta \cos^{2}\left(\theta \right)+\gamma }{\sin^{3}\left(\theta \right)}$$

The filter coefficients α, β and γ are calculated as:

(7) $$\begin{array}{c} \alpha =\frac{\left(z^{2}z_{e}^{2}-2z^{2}z_{e}z_{o}-z^{2}z_{o}^{2}+4z^{2}-4zz_{e}-4zz_{o}+z_{e}^{2}+2z_{e}z_{o}-z_{o}^{2}\right)}{\left(4\left(z_{e}-z_{o}\right)z^{2}\right)} \end{array}$$

(8) $$\begin{array}{c} \beta =\frac{\left(2z^{2}z_{e}^{2}-2z^{2}z_{o}^{2}+8z^{2}+4zz_{e}-4zz_{o}+z_{e}^{2}-2z_{e}z_{o}+z_{o}^{2}\right)}{\left(4\left(z_{e}-z_{o}\right)z^{2}\right)} \end{array}$$

(9) $$\begin{array}{c} \gamma =\frac{\left(z^{2}z_{e}^{2}+2z^{2}z_{e}z_{o}-z^{2}z_{o}^{2}-4z^{2}\right)}{\left(4\left(z_{e}-z_{o}\right)z^{2}\right)} \end{array}$$

Equation (4) delineates the filtering function associated with the topology depicted in Figure 1. The form of this filtering function aligns with the structure of type 1 Chebyshev polynomials, facilitating the extraction of pertinent electrical parameter values. However, the presence of a frequency-dependent term within the denominator of the filtering function contravenes the anticipated equal ripple attributes of the Chebyshev polynomial. This deviation necessitates a strategic refinement of the filtering function, an approach that is effectively showcased.

In pursuit of rectifying this deviation, a pivotal step involves a meticulous normalization process applied to the filtering function. This normalization step serves to curtail the influence of frequency variations, thereby restoring the characteristic equal ripple behavior. The resultant ripple factor, an outcome of this normalization, serves as a critical metric. Subsequently, leveraging this ripple factor, a synthesized rendition of the filtering function is systematically engineered, enabling the identification of an optimal solution that harmonizes with the desired filtering criteria.

(10) $$G\left(\theta \right)=\varepsilon \frac{\tilde{G}\left(\theta \right)}{\tilde{G}\left(\theta_{Norm}\right)}$$

where θ denotes the normalized electrical length and $\tilde{G}\left(\theta \right)$ is the normalization factor and is driven as:

(11) $$\tilde{G}\left(\theta \right)=\frac{\cos^{4}\left(\theta \right)+\sigma \cos^{2}\left(\theta \right)+\varsigma }{\sin^{3}\left(\theta \right)}$$

where

(12) $$\alpha =\frac{\varepsilon }{\tilde{G}\left(\theta_{Norm}\right)}$$

(13) $$\sigma =\frac{\beta }{\alpha }$$

(14) $$\varsigma =\frac{\gamma }{\alpha }$$

The return loss is correlated with the ripple factor ε by

(15) ε=(10LRLR1010−1)−1/2

By using the transfer function, the value of the parameters α, β, and γ may be determined from Equation (8).

(16) $${\left|S_{21}\left(j\omega \right)\right|}^{2}=\frac{1}{1+\varepsilon^{2}{\left|\frac{\tilde{G}\left(\theta \right)}{\tilde{G}\left(\theta_{Norm}\right)}\right|}^{2}}$$

This complies with the following characteristics: UWB bandwidth (4.5–10 GHz), four transmission poles within the passband (for better selectivity), equal ripple (approximating Chebyshev frequency response), adjustable ripple level by ripple factor ε. The first derivatives can be used to determine the transfer function’s relative maxima and minima. To produce the filtering coefficients, Suppose the denominator of Equation (2) is equal to *H*.

(17) $$H=1+G^{2}\left(\theta \right)$$

By using Equation (8) and applying the chain rule, the derivative reflection coefficient is found as:

(18) $$\frac{d}{d(\theta )}{\left|S_{11}\left(j\omega \right)\right|}^{2}=\frac{1}{G^{2}}\times \frac{2\varepsilon^{2}\tilde{G}\left(\theta \right)}{{\tilde{G}}^{2}\left(\theta_{Norm}\right)}\times \frac{d\tilde{G}\left(\theta \right)}{d(\theta )}$$

The disappearance of $H^{-1}$ relates to the transmission poles at θ=0 and at π. However, the disappearance of $\tilde{G}\left(\theta \right)$ relates with the transmission poles located at

(19) $$\theta_{z}^{\left(\pm ,\pm \right)}=\arccos\left(\pm \frac{1}{2}\sqrt{-2\sigma \pm 2\sqrt{\sigma^{2}-4}\varsigma }\right)$$

To ensure that the transfer function has four poles, it is essential that

(20) σ<0ς>0

To address the disparities

(21) $$\sigma^{2}-4\varsigma >0$$

(22) σ+ς=1>0

whereas θ∈(0,π). We determine the position of the transmission poles as $\theta^{\left(+,+\right)}$, $\theta^{\left(+,-\right)}$, $\theta^{\left(-,-\right)}$, $\theta^{\left(-,+\right)}$, respectively. The frequencies of the ripple peaks may be calculated using

(23) $$\frac{d\tilde{G}\left(\theta \right)}{d(\theta )}=\frac{[3\cos^{4}\left(\theta \right)+\left(\sigma -4\right)\cos^{2}\left(\theta \right)-\left(2\sigma +\varsigma \right)]\cos\left(\theta \right)}{\sin^{6}\left(\theta \right)}$$

In Equations (21) and (22), the domain defined for all σ and ς can be defined as $\cos(\theta_{pk}^{\pm ,+})>1$. Therefore, the matching peaks in the ripples for these solutions do not exist and are ignored. $\theta_{pk}^{{}^{\left(+,-\right)}}$ and $\theta_{pk}^{{}^{\left(-,-\right)}}$ are the remaining roots, and they are equivalent to the passband’s first and third ripple peaks, in that order. Equal-ripple may be imposed by matching the filtering function at the cut-off frequency with the first and second peaks.

(24) $$\tilde{G}\left(\theta_{L}\right)=-\tilde{G}\left(\theta_{Pk}^{\left(+,-\right)}\right)=\tilde{G}\left(\frac{\pi }{2}\right)$$

where the lower cutoff frequency is denoted by the angular $\theta_{L}$. By solving this set of simultaneous Equations (21) and (22), we obtain

(25) $$\sigma =\frac{3}{4}{\left[\sin\left(\frac{BW}{2}\right)+\frac{1}{3}\right]}^{2}$$

(26) $$\varsigma =\frac{1}{4}\cos^{2}\left(\frac{BW}{2}\right)\left[1-\sin\left(\frac{BW}{2}\right)\right]$$

Here, BW represents the bandwidth. In this specific instance, the normalizing factor equals ς and from Equation (10)–(12) γ is equal to ε.

(27) $$\tilde{G}\left(\theta_{Norm}=\frac{\pi }{2}\right)=\varsigma$$

(28) γ=ε

**Figure 3:**
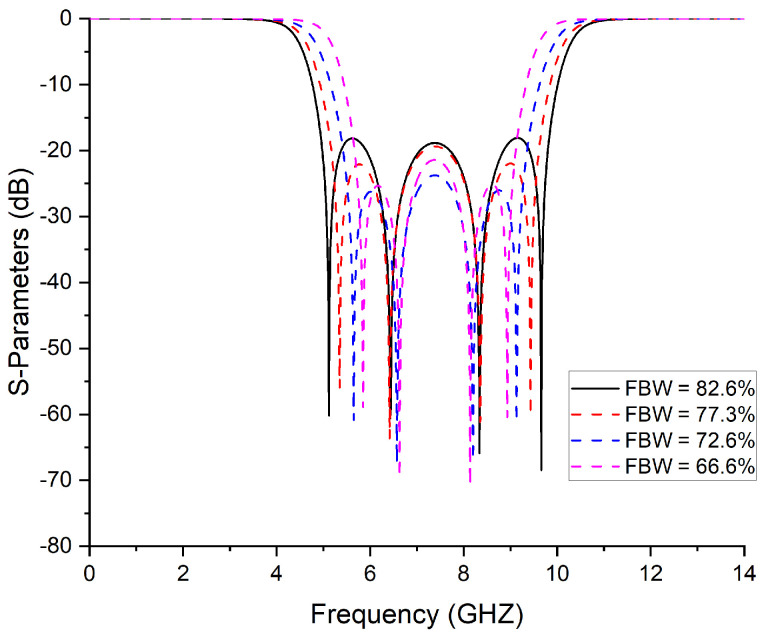
Change in FBW of 4th-order bandpass filter by changing impedances.

The impedance values for various bandwidths are calculated in Table 1 and shown in Figure 3. A line calculator using a freely available tool is used to calculate width, length, and gaps. The fourth-order filter was fabricated using the parameters given in the table: *z* = 28.57, $z_{e}$ = 83.8, $z_{o}$ = 172.9, and FBW = 82.6.

**Figure 4:**
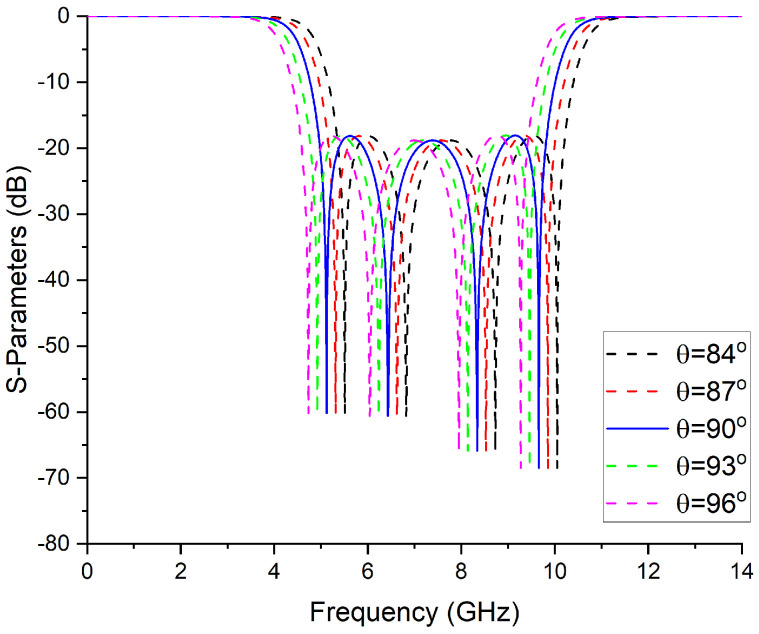
Frequency response with a variable electrical length.

By altering the electrical length, it is evident that one can exercise control over the center frequency (CF). As depicted in Figure 4, an increase in the value of θ results in a downward shift of the CF towards lower frequencies. Conversely, a decrease in the value of θ causes the CF to ascend towards higher frequencies. However, it is worth noting that the maneuverability of transmission zeros is influenced by their proximity to the resonant frequencies. Specifically, adjusting the resonant frequencies of circuit elements can induce a limited displacement of the transmission zeros, thereby offering a degree of control.

### 2.2. Synthesis of Seventh-Order Bandpass Filter

**Figure 5:**
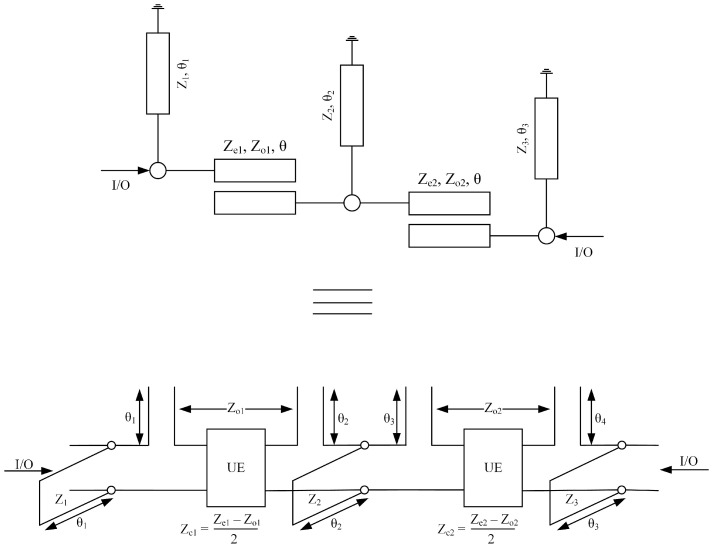
Schematic of 7th-order UWB-BPF with its equivalent circuit model.

This section introduces a comprehensive configuration aimed at substantiating the proposed synthesis approach for a seventh-order bandpass filter. The structure in question entails a pair of quarter-wavelength coupled lines, each adorned with three short-circuited stubs characterized by an impedance value denoted as Z and an electrical length parameter θ. It is noteworthy that the coupled lines exhibit distinct even and odd mode characteristic impedances, represented as $Z_{e}$ ohms and $Z_{o}$ ohms, respectively. In the endeavor to synthesize the seventh-order ultra-wideband bandpass filter (seventh-order UWB-BPF), the underlying distributed equivalent circuit model illustrated in Figure 5 assumes a pivotal role. This model serves as the cornerstone for the extraction of the pertinent filtering function, instrumental in guiding the synthesis process.

(29) $$\begin{array}{c} \left[T\right]=\prod_{q=1}^{3}\left(\begin{array}{cc} 1 & 0\\ \frac{1}{jz\tan(\theta )} & 1 \end{array}\right)\times \prod_{q=1}^{2}\left(\begin{array}{cc} \cos(\theta ) & jz_{c}\sin\left(\theta \right)\\ \frac{j\sin(\theta )}{z_{c}} & \cos(\theta ) \end{array}\right)\times \prod_{q=1}^{4}\left(\begin{array}{cc} 1 & \frac{-jz_{o}}{\tan(\theta )}\\ 0 & 1 \end{array}\right) \end{array}$$

(30) $$G_{7}\left(\theta \right)=\frac{A\cos^{7}\left(\theta \right)-B\cos^{5}\left(\theta \right)+C\cos^{3}\left(\theta \right)-D\cos\left(\theta \right)}{\sin^{5}\left(\theta \right)}$$

(31) $$\begin{array}{c} A=\frac{1}{4}\left(-z_{o}+4z-z_{e}\right)\left(zz_{e}+zz_{o}+2z-z_{e}-z_{o}\right)\left(zz_{e}+zz_{o}-2z+z_{e}+z_{o}\right)\left(z_{o}+z_{e}\right)\\ \;\times {\left(\frac{1}{2}{\left(z_{e}-z_{o}\right)}^{2}z^{3}\right)}^{-1} \end{array}$$

(32) $$\begin{array}{c} B=-3\left(z_{e}^{2}+\frac{2}{3}z_{e}z_{o}+z_{o}^{2}-4\right)\left(z_{o}+z_{e}\right)z^{3}+(\frac{1}{4}(3z_{e}^{4}+4z_{e}^{3}z_{o}+\left(2z_{o}^{2}-48\right)z_{e}^{2}+\\ \;\left(4z_{o}^{3}-64z_{o}\right)z_{e}+3z_{o}-48z_{o}^{2})z^{2}+(4\left(z_{o}+z_{e}\right)\left(z_{e}^{2}-z_{o}^{2}\right)z-\frac{1}{2}{\left(z_{e}-z_{o}\right)}^{2}{\left(z_{o}+z_{e}\right)}^{2}\\ \;\times \;{\left(\frac{1}{2}{\left(z_{e}-z_{o}\right)}^{2}z^{3}\right)}^{-1} \end{array}$$

(33) $$\begin{array}{c} C=-3\left(z_{e}^{2}+\frac{2}{3}z_{e}z_{o}+z_{o}^{2}-4\right)\left(z_{o}+z_{e}\right)z^{3}+(\frac{1}{4}(3z_{e}^{4}+4z_{e}^{3}z_{o}+\left(2z_{o}^{2}+36\right)z_{e}^{2}+(4z_{o}^{3}-\\ \;8z_{o})z_{e}+3z_{o}^{4}-36z_{o}^{2})z^{2}-(2\left(z_{o}+z_{e}\right){\left(z_{e}-z_{o}\right)}^{2}z-\frac{1}{4}{\left(z_{e}-z_{o}\right)}^{4}\\ \;\times {\left(\frac{1}{2}{\left(z_{e}-z_{o}\right)}^{2}z^{3}\right)}^{-1} \end{array}$$

(34) $$\begin{array}{c} D=z^{2}\left(-z_{o}+2z_{e}\right)\left(z_{o}+z_{e}\right)\left(-z_{o}-2+z_{e}\right)z-\left(\frac{1}{4}\left(z_{e}^{2}-2z_{e}z_{o}+z_{o}^{2}-8\right)\right){\left(z_{e}-z_{o}\right)}^{2}\\ \;\times \;{\left(\frac{1}{2}{\left(z_{e}-z_{o}\right)}^{2}z^{3}\right)}^{-1} \end{array}$$

In this case, $z_{e},z_{o}>0$, the provided Equation (30) demonstrates that a total of seven transmission poles can be achieved in the passband by appropriately adjusting the filter’s coefficient values. However, it is seen that one of the seven transmission poles is positioned at a phase angle of $90^{\circ }$ relative to the central frequency. An ideal equi-ripple frequency response in a filter is attained by strategically positioning the transmission zeros and poles using an appropriate filter design technique. Nevertheless, the presence of frequency-dependent elements in the filtering function’s denominator prevents the filter from achieving an ideal response. This is due to the distortions introduced by these elements, which cannot be fully compensated for by the placement of transmission zeros and poles alone. Therefore, the authors have modified the filter’s transfer function coefficients to minimize the influence of the frequency-dependent element and enhance the filter’s frequency response to the greatest extent possible. The synthesis technique described in the preceding section was applied to the filtering function given in Equation (28) to determine the appropriate coefficient values. The same procedures were followed, beginning with evaluating the roots of the filtering function. The passband location of the ripple peak Was determined using the first derivative of the filtering function.

## 3. Results

**Figure 6:**
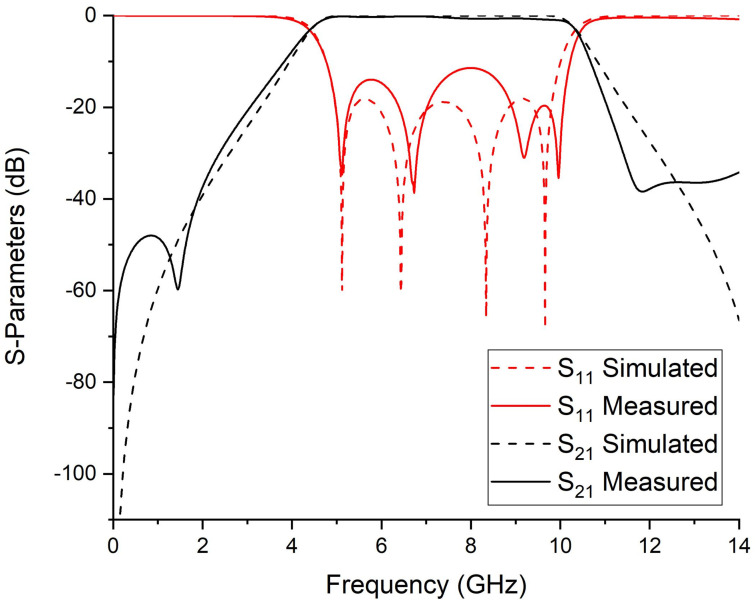
Simulated and measured S-parameter response of 4th-order UWB-BPF.

**Figure 7:**
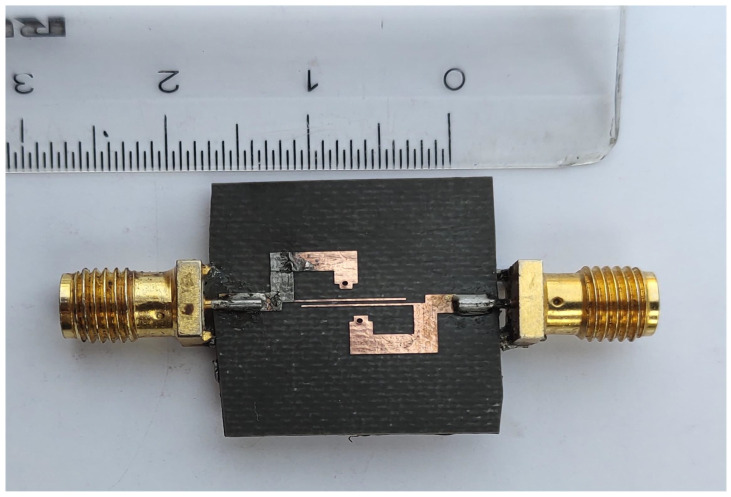
Fabricated prototype of 4th-order UWB-BPF.

To validate the novel synthesis of the fourth-order ultra-wideband bandpass filter, microstrip transmission lines are used to design the proposed UWB-BPF filter. The electrical parameters are determined by the proposed synthesis procedure. For the passband with the bandwidth of 5.5 GHz centered at 7.25 GHz, the values of σ and ς are 0.248 and 0.235, respectively. By using Equations (10)–(13), we found the Chebyshev polynomials which are α=184.74, β=207.2, and γ=22.26, whereas the ripple level calculated for 20 dB is ε=0.1. The characteristic impedance found by solving Equations (5)–(7) are Z=28.57, $Z_{o}=83.8$, and $Z_{e}=172.9$ (unit in Ohm). After finding the electrical parameters, the physical parameters are determined, which are $W_{c}=0.2$, $W_{s}=2.16$, $W_{in}=0.9$, $L_{s}=2.30$, $L_{c}=7.2$, and $L_{in}=2.0$ (unit in mm). The proposed UWB bandpass filter is fabricated on RT Duroid 5880 with ($\varepsilon_{r}=2.2$, tanδ=0.0009 and height h=0.787 mm). The S-parameter response and fabricated prototype of fourth-order UWB-BPF is shown in Figure 6 and Figure 7. Four transmission poles at 4.91, 6.85, 9.22, and 10.18 GHz are measured. The simulated and measured results show good agreement.

**Figure 8:**
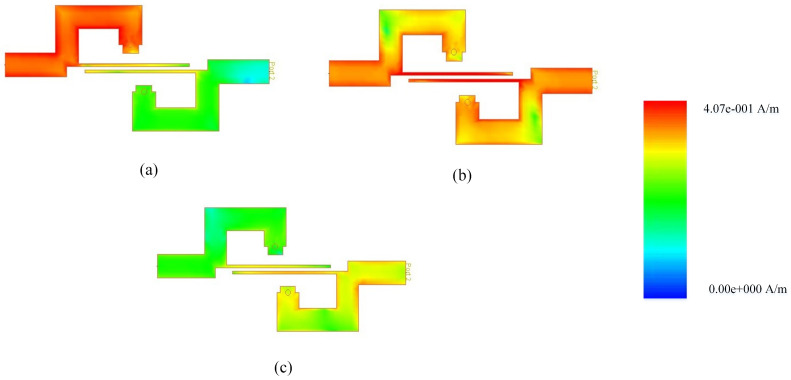
(**a**–**c**) Current distribution of 4th-order UWB-BPF.

The current distribution at 1.9 GHz, 7.25 GHz, and 11.75 GHz is illustrated in Figure 8. Figure 8b reveals that the single-stage couple line significantly influences at passband frequency, while at the band stop frequencies, the active components are the loaded stubs, as evident in Figure 8a,c.

**Figure 9:**
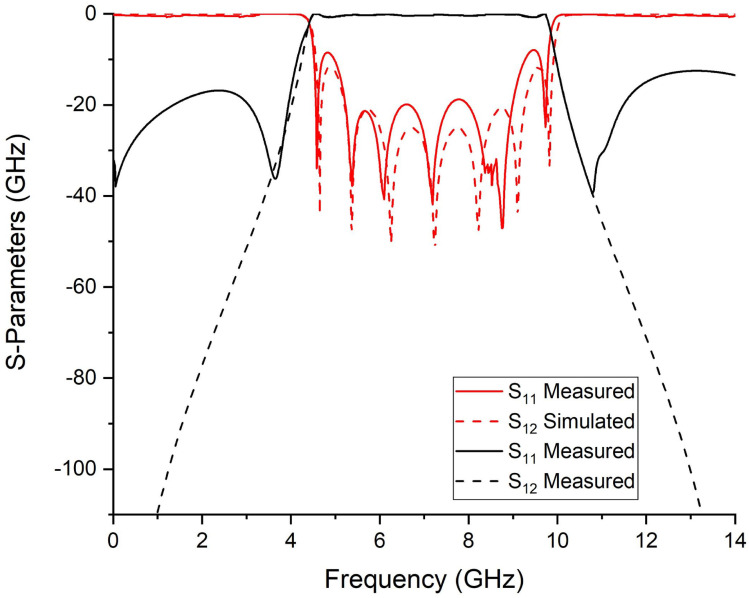
Simulated and measured S-parameter response of 7th-order UWB-BPF.

**Figure 10:**
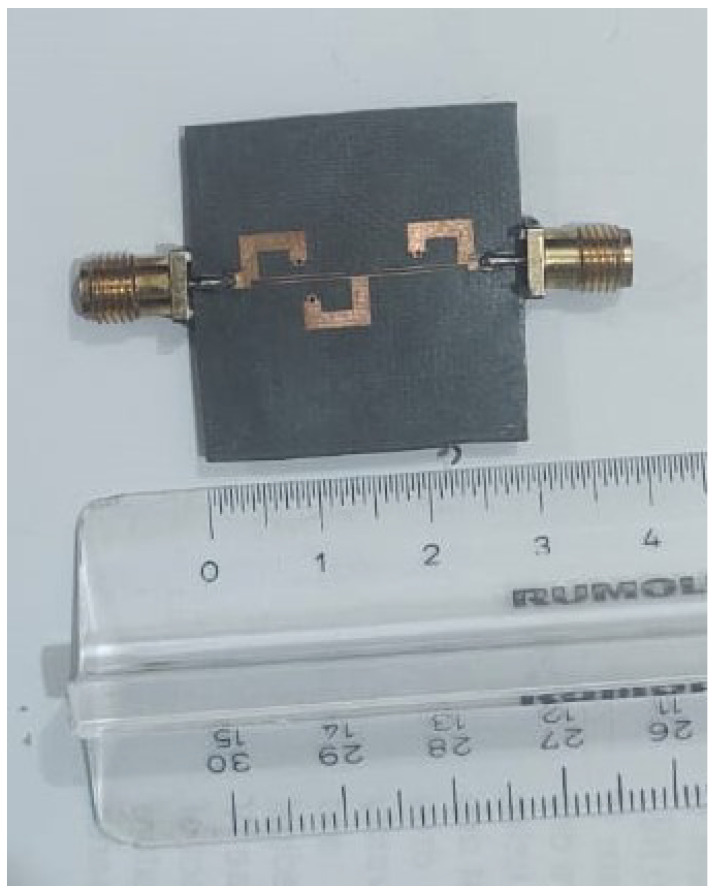
Fabricated prototype of 7th-order UWB-BPF.

Figure 9 and Figure 10 show the S-parameter response of seventh-order UWB-BPF and its fabricated prototype. By increasing the order of the proposed UWB-BPF, seven transmission poles were measured at 4.67, 5.4, 6.29, 7.28, 8.27, 9.15, and 9.88 GHz. A fractional bandwidth of 82.6% at 7.25 GHz is attained with a minimal return loss of 11.8 dB. The measured out-of-band rejection is greater than 12.9 dB from 10 GHz to 14 GHz. And, only one transmission zero is measured. Measured results may exhibit the presence of extra transmission zeros that are not observed in the simulated results. This phenomenon can be attributed to various factors, such as the impact of bending and soldering, which can modify the electrical characteristics of the filter components and impact the overall performance of the filter. Moreover, coupling between the transmission lines can generate additional transmission zeros, which may not be precisely accounted for in the simulations. PCB via holes introduces parasitic inductance and capacitance, which cause the little frequency shift. In the proposed filter, the FCC requirements are fulfilled, although the out-of-band characteristics of theoretical and measured filters are different.

**Figure 11:**
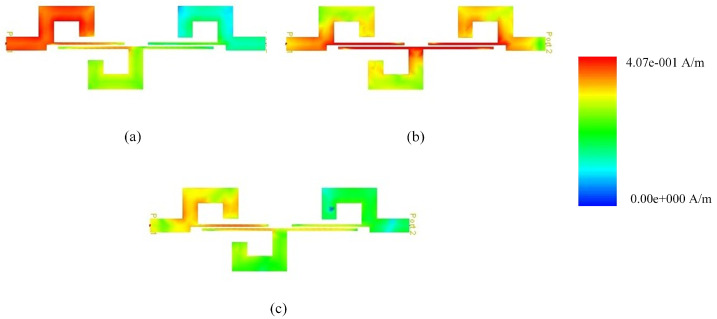
(**a**–**c**) Current distribution of 7th-order UWB-BPF.

The physical parameters $W_{c}=0.2$, $W_{s1}=W_{s3}=2.16$, $W_{s2}=2.16$, $L_{s1}=L_{s2}=L_{s3}=2.30$, $L_{c}=7.2$ are found by using the electrical parameters which are $z_{s1}=z_{s3}=30.8$, $z_{s2}=16.4$, $z_{e}=186$ and $z_{o}=93$. Whereas $z_{s}$ and $W_{s}$ represent the impedance and width of loaded short circuit stubs, $z_{e},z_{o}$ and $W_{c}$ represent the even/odd impedance and width of coupled lines. Moreover, the $l_{s}$ and $L_{c}$ represent the physical lengths of loaded stubs and couple lines, respectively. During circuit modeling, the impacts of substrate losses are mitigated using the quasi-Newton and pattern search optimization approaches. The current distribution at 3.45 GHz, 7.28 GHz, and 11.35 GHz is illustrated in Figure 11. Figure 11b reveals that the single-stage couple line significantly influences the passband frequency, while at the band stop frequencies, the active components are the loaded stubs, as evident in Figure 11a,c.

**Figure 12:**
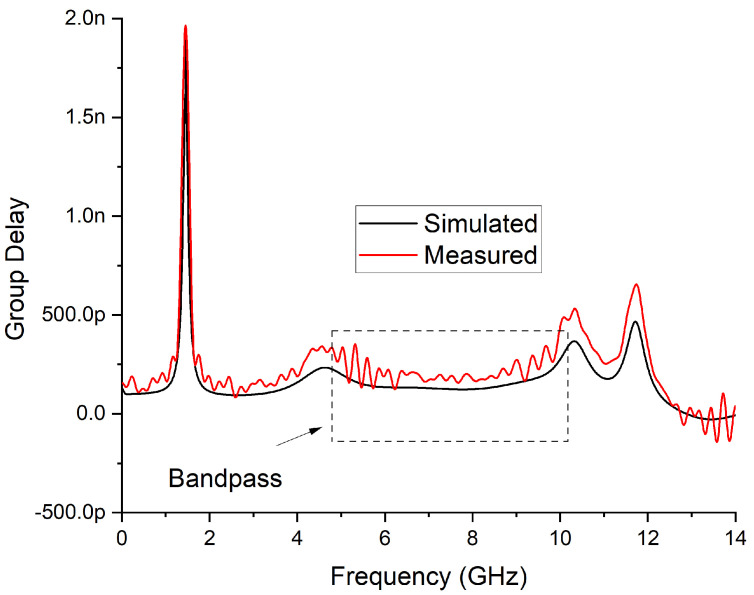
Measured group delay of proposed 7th-order UWB-BPF.

Figure 12 shows the group delay of the proposed seventh-order UWB-BPF. The maximum group delay within the passband is not greater than 354 ps. However, there are several factors that can cause variation in group delay within the passband of a filter. Some of the most common factors include the filter’s order, the location and spacing of its poles, and the magnitude and phase response of its transfer function. Other factors that can affect the group delay include the filter’s passband ripple, the slope of its transition band, and the frequency response of the filter’s components. In general, the goal of filter design is to minimize variation in group delay within the passband while achieving the desired frequency response characteristics.

Table 2 provides a concise summary of the comparison with previously established UWB BPFs. In contrast to other designs of a similar size, the suggested UWB bandpass filter has a small size, higher electrical performance, and a reasonably high fractional bandwidth. The suggested design’s simplicity and ease of fabrication are also obvious. The manufactured prototype of the suggested UWB BPF is illustrated in Figure 2.

## 4. Conclusions

This paper introduces an innovative approach for the synthesis of the proposed ultra-wideband (UWB) bandpass filter, showcasing its distinctive characteristics. The underpinning methodology leverages a distributed equivalent circuit model, instrumental in attaining the sought-after quasi-Chebyshev filtering response. By skillfully mapping this quasi-Chebyshev-type filtering function onto the established Chebyshev type-I filtering response, the essential filter parameters are rigorously ascertained. The resulting UWB bandpass filter design stands out due to its compact form factor, effectively addressing size constraints. To substantiate the efficacy of the design procedure proposed herein, the envisioned filter is realized through fabrication. The subsequent comparative analysis of the simulated and measured outcomes underscores a noteworthy alignment, affirming the robustness and viability of the presented design methodology.

## Figures and Tables

**Table 1:** Synthesized values of filter parameters shown in Figure 2 for various bandwidths.

FBW	Return Loss	*z*	$z_{o}$	$z_{e}$
**(%Age)**	**(dB)**	**(Ohm)**	**(Ohm )**	**(Ohm)**
56	10	14.82	122.33	206.97
61.3	10	17.40	114.65	198.73
66.6	10	20.76	109.81	191.36
72.6	10	23.30	102.65	185.64
77.3	10	25.36	91.36	179.64
82.6	10	28.51	83.8	172.95

**Table 2:** Comparison of the fabricated prototypes.

Ref	CF’s	TP’s	RL’s	IL	Circuit Size	FBW
	**(GHz)**		**(dB)**	**(dB)**	$\left(\lambda_{g}\times \lambda_{g}\right)$	**(%Age)**
[1]	0.9	4	>20	0.76	0.26×0.26	50
[2]	6.85	6	>10	0.81	0.68×0.35	78.8
[3]	6.85	6	>10	N/A	0.69×0.18	111
[4]	8	3	>15	>0.5	0.48×0.15	126
[5]	2.5	4	>15	>0.1	N/A	53
[6]	7.03	7	>20	N/A	2.14×0.53	112.2
[18]	6.95	4	>15	>N/A	1.02×0.51	113.6
[19]	3.65	2	>11.3	0.9	0.53×0.02	156
[20]	3.05	10	>11	0.5	1.09×0.16	142
This work	7.25	7	>11.8	0.15	0.29×0.37	** 82.6 **

## Data Availability

Data supporting this paper’s findings are available upon reasonable request, subject to applicable data sharing agreements and ethical considerations.
